# Uterine smooth muscle tumor of uncertain malignant potential: a case report

**DOI:** 10.3389/fonc.2026.1826552

**Published:** 2026-05-20

**Authors:** Xiujuan Chen, Fei Chen, Yiwen Ding, Wenzhe Xu, Xiaoguang Huo, Xiaojiao Meng

**Affiliations:** Department of Ultrasound, Zibo Central Hospital, Zibo, Shandong, China

**Keywords:** magnetic resonance imaging diagnosis, pathological diagnosis, surgical treatment, ultrasound diagnosis, uterine smooth muscle tumor of uncertain malignant potential

## Abstract

Uterine smooth muscle tumor of uncertain malignant potential (STUMP) is a rare borderline smooth muscle tumor. This paper reports a case of STUMP and discusses its clinical characteristics, diagnostic challenges, and treatment strategies in combination with relevant literature. The patient was a young female who presented with menorrhagia and severe anemia. Pelvic ultrasound revealed a large mass in the uterus, which was suspected to be a uterine leiomyoma. Surgical resection of the mass was subsequently performed, and postoperative pathological examination confirmed the diagnosis of STUMP. The risk factors and biological mechanisms underlying the development of STUMP remain poorly understood. The tumor lacks specific clinical and imaging features, making diagnosis primarily dependent on histopathological evaluation. Surgery is the mainstay of treatment, and close follow-up is recommended postoperatively.

## 1 Introduction

Uterine smooth muscle tumor of uncertain malignant potential (STUMP) is a borderline or intermediate smooth muscle tumor that falls between benign uterine leiomyoma and malignant uterine leiomyosarcoma (LMS). STUMP is a rare tumor, typically occurring in patients aged 40–50 years (1–3) and accounting for approximately 2%-5% of all uterine smooth muscle tumors (4). It grows slowly, with a 5-year survival rate of 90%-100% and a recurrence rate of 7.3%-36.4% (1, 5–7). The tumor may recur as STUMP or progress to LMS.

In clinical practice, the diagnosis of STUMP is challenging due to the lack of specific clinical and imaging features. Pelvic ultrasound is the most commonly used initial imaging modality. The diagnosis relies primarily on pathological examination, and the histopathological diagnostic criteria are inherently subjective. Immunohistochemistry and molecular testing can provide valuable adjunctive information for the diagnosis (8).

Hysterectomy is recommended for patients who have no desire to preserve fertility. For those who wish to preserve fertility, local resection of the STUMP lesion may be performed after obtaining fully informed consent and conducting a thorough risk assessment. Hysterectomy is recommended following completion of childbearing. Regular follow-up for at least 5 years is recommended postoperatively (8, 9).

This paper reports a case of STUMP and reviews the relevant literature to enhance the understanding of this disease and improve diagnostic accuracy, thereby reducing the risk of misdiagnosis.

## 2 Case report

This study received approval from the Ethics Committee of Zibo Central Hospital, under ethics number 2025 Yan No. 225.

A 38-year-old female was admitted to our hospital due to menorrhagia for 2 years and a suspected uterine leiomyoma detected during a physical examination 6 months prior. Two years prior, she developed menorrhagia without an apparent trigger, with bleeding approximately three times her usual volume, accompanied by blood clots. Her menstrual cycle and duration remained unchanged, as did her dysmenorrhea. She also noted increased leukorrhea, but denied frequent urination, urgency, or dysuria. She did not seek further medical evaluation or treatment at that time.

Six months ago, during a physical examination at a health screening facility, pelvic ultrasonography revealed multiple uterine fibroids, with the largest measuring 103 mm × 68 mm. Routine blood test showed hemoglobin 50 g/L, and the patient experienced generalized fatigue and headache. She was advised to visit a higher-level hospital for further evaluation of the cause of anemia and appropriate management, but she did not seek further medical care. The patient subsequently presented to our hospital due to aggravated fatigue.

**Figure 1:**
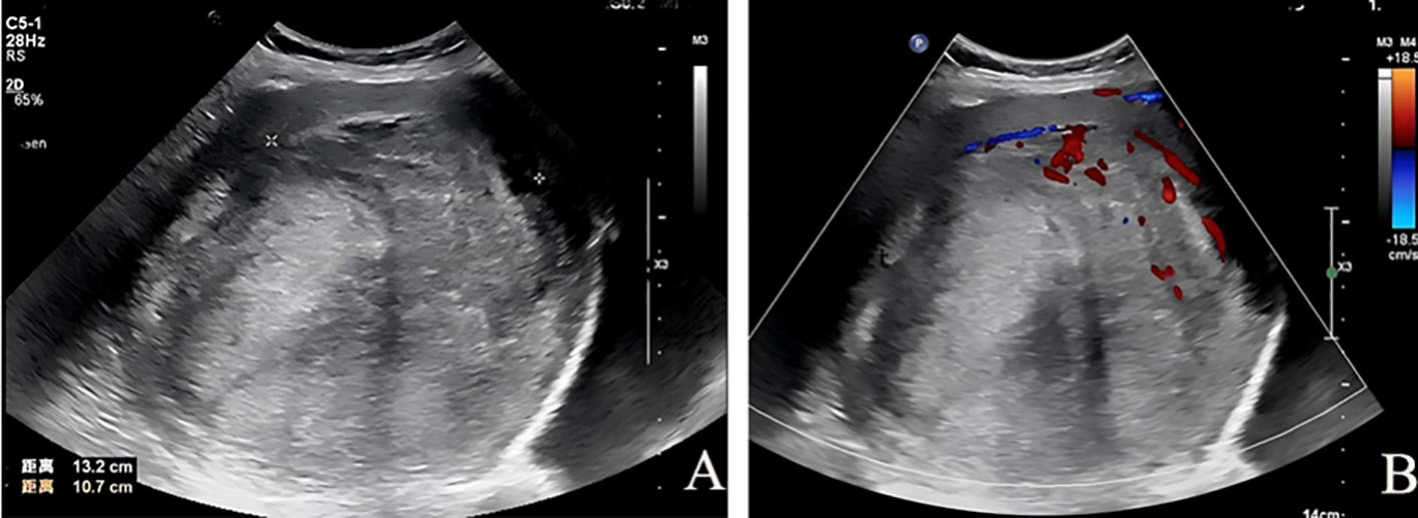
Pelvic ultrasound images. **(A)** A mixed echogenicity mass measuring 132 mm × 107 mm is observed in the uterus, with a clear boundary and heterogeneous internal echogenicity. **(B)** Color Doppler Flow Imaging (CDFI) shows scant intratumoral blood flow and a semi-annular blood flow signal surrounding the mass.

Transabdominal pelvic ultrasound showed an anteverted uterus that was significantly enlarged and globular in shape. The endometrium was not clearly delineated. A mass of mixed echogenicity, measuring approximately 132 mm×107 mm, was detected in the uterus (**Figure 1A**). It had a clear boundary and heterogeneous internal echoes. Color Doppler Flow Imaging (CDFI) demonstrated a small amount of blood flow inside the mass and a semi-annular blood flow signal around it (**Figure 1B**). No abnormalities were found in the bilateral adnexa. The ultrasound suggested a mixed echogenicity uterine mass, possibly a uterine leiomyoma.

**Figure 2:**
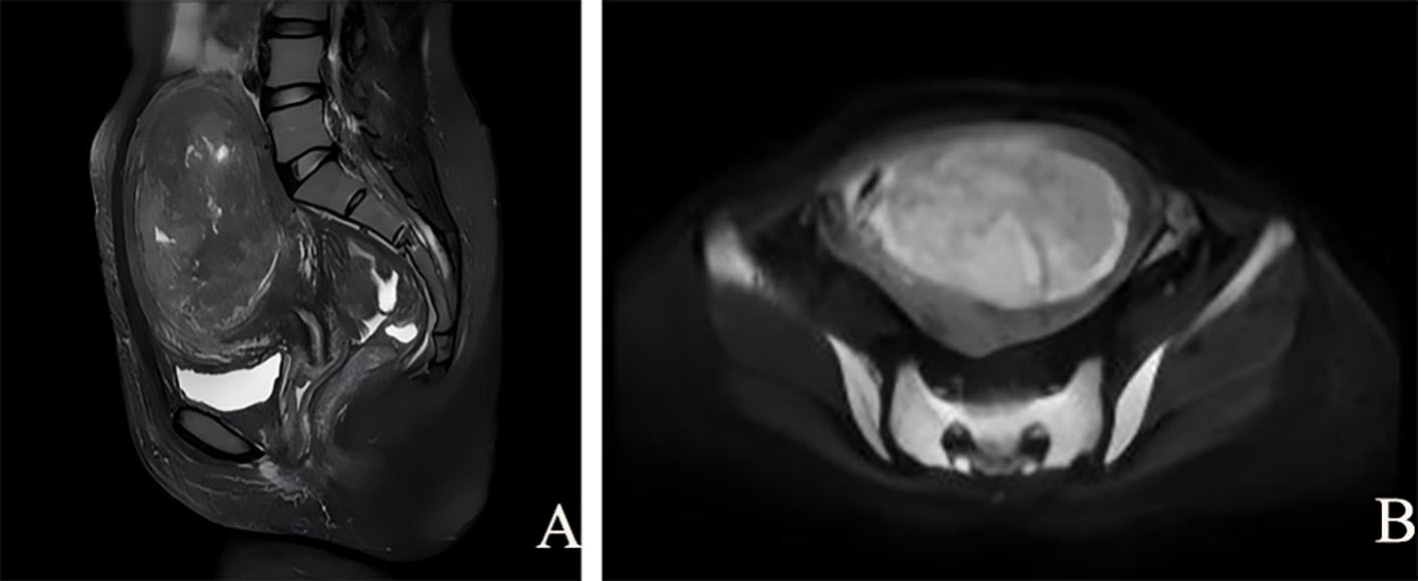
Pelvic magnetic resonance imaging (MRI). **(A)** Sagittal T2-weighted imaging (T2WI) demonstrates a large mass lesion within the uterine myometrium. The lesion shows intermediate-to-low signal intensity on T2WI, with heterogeneous areas of slightly hyperintense and markedly hyperintense signal. **(B)** Diffusion-weighted imaging (DWI) shows mildly hyperintense signal within the lesion.

Pelvic magnetic resonance imaging (MRI) showed a markedly enlarged uterus. A large space-occupying lesion measuring approximately 11.8×7.9 cm is identified within the uterine myometrium. The lesion demonstrates isointense signal on T1-weighted imaging (T1WI) and heterogeneous moderately to markedly hyperintense signal on T2-weighted imaging (T2WI) with cystic changes (**Figure 2A**). On diffusion-weighted imaging (DWI), the lesion has ill-defined margins and is predominantly slightly hyperintense (**Figure 2B**), with focal reduced signal on apparent diffusion coefficient (ADC) mapping.

All eight tumor markers (including human chorionic gonadotropin, carcinoembryonic antigen, alpha-fetoprotein, neuron-specific enolase, carbohydrate antigen CA-125, carbohydrate antigen CA15-3, carbohydrate antigen CA19-9, and carbohydrate antigen 72-4) were within normal limits, and human epididymis protein 4 was also normal. A routine blood test showed hemoglobin 46 g/L. Gynecological examination was not performed as the patient reported no history of sexual activity.

**Figure 3:**
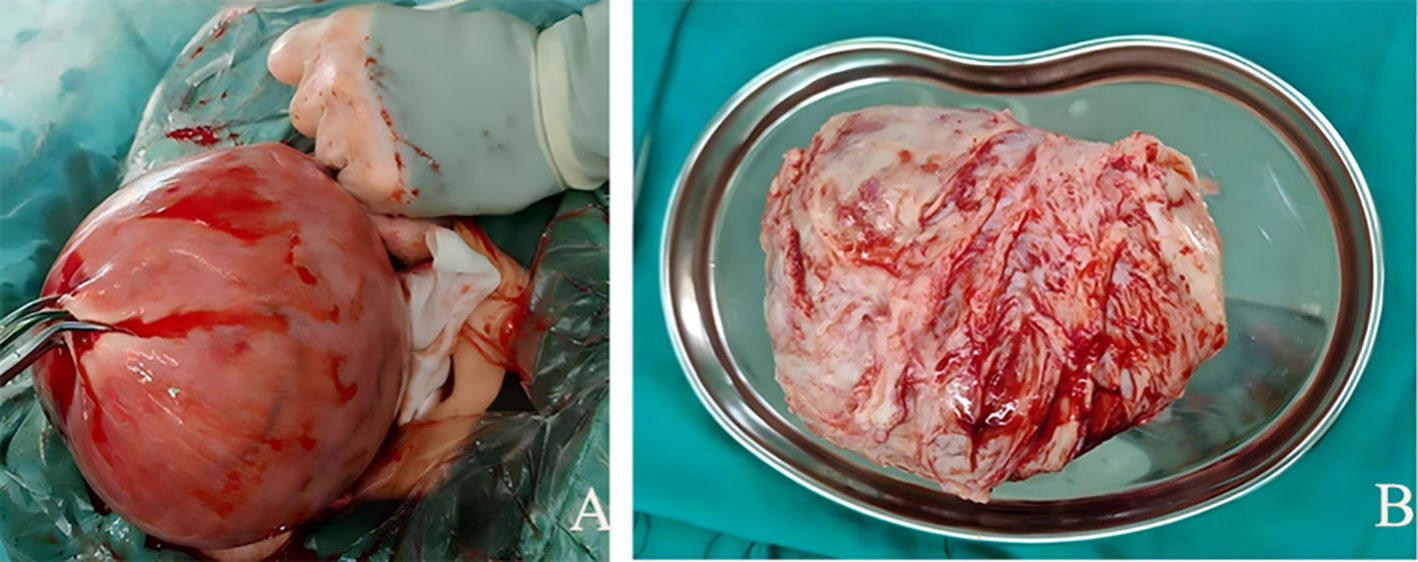
Intraoperative images of the mass. **(A)** A large mass measuring approximately 13 cm × 11 cm is observed on the posterior uterine wall. **(B)** The cut surface is partially nodular and whorled.

Due to severe anemia resulting from large uterine fibroids, the patient received a transfusion of 4 units of leukocyte-reduced red blood cells. A repeat complete blood count showed that the hemoglobin level had increased to 72 g/L, after which transabdominal myomectomy was performed. Intraoperatively, a mass measuring approximately 13 cm×11 cm was observed on the posterior uterine wall (**Figure 3A**). The cut surface was grayish-white, partially nodular and whorled, with a firm consistency (**Figure 3B**).

**Figure 4:**
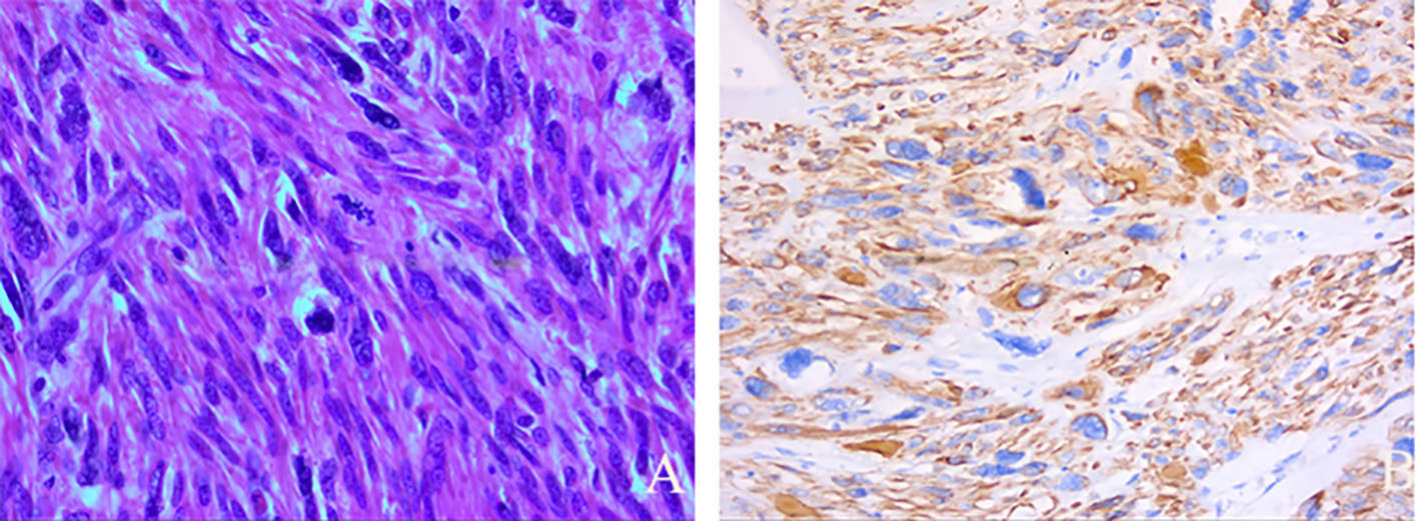
Pathological and immunohistochemical images. **(A)** Hematoxylin and Eosin staining shows a mixture of spindled and epithelioid tumor cells with large, hyperchromatic nuclei. Multinucleated giant cells and bizarre cells are present. Mitotic figures are present, but at a rate of fewer than 2 per 10 high-power fields (HPF) (at 400× magnification). **(B)** Desmin immunohistochemistry shows cytoplasmic positivity in tumor cells (at 200× magnification).

Pathological diagnosis: The tumor (submucosal and intramural) showed diffuse proliferation of bizarre, syncytial, and multinucleated giant tumor cells of varying sizes. These cells contained abundant eosinophilic cytoplasm. Mitotic count was <2 per 10 high-power field (HPF) (**Figure 4A**). No obvious necrosis was found. The tumor showed expansive growth. Combined with immunohistochemistry (**Figure 4B**) and clinical findings, a diagnosis of huge atypical smooth muscle tumor with bizarre nuclei (STUMP) was made.

Immunohistochemistry revealed the following profile: Smooth Muscle Actin (SMA): (+); Desmin: (+); cluster of differentiation 10 (CD10): Diffusely (+); CD34: Positive in vascular endothelium; Fumarate Hydratase (FH): (+); Succinate Dehydrogenase Complex Iron Sulfur Subunit B (SDHB): (+); Estrogen Receptor (ER): (++), positive in 3% of cells; Progesterone Receptor (PR): (+++), positive in 90% of cells; Human Melanoma Black 45 (HMB-45): (−); Melan-A: (−); signal transducer and activator of transcription 6 (STAT6): (−); retinoblastoma 1 (RB1): (−); p53: (−); Ki-67: (+), positive in 3%-12% of cells.

The pathological slides were reviewed by a higher-level hospital, which confirmed the diagnosis. The patient was fully informed of the pathological diagnosis, the biological behavior of STUMP, and the potential risk of recurrence. Hysterectomy was recommended as a further prophylactic procedure. However, the patient firmly declined further surgery due to a strong desire to preserve fertility. After comprehensive discussion and acquisition of informed consent, a strategy of intensive surveillance was adopted instead. Postoperative ultrasound examinations at 1, 3, and 6 months revealed no abnormal findings. At 1 month postoperatively, hemoglobin was 97 g/L (mild anemia), rising to 124 g/L (normal range) at 3 months. The patient remains under regular follow-up. The clinical course and key management steps of the patient are summarized in the timeline (**Table 1**).

**Table 1:** Timeline of clinical events for the patient with STUMP.

Date (YYYY-MM-DD)	Clinical event
2023-03-01	Menorrhagia (3× normal volume, with clots) and increased leukorrhea; no medical care
2024-11-03	Ultrasound: multiple uterine leiomyomas (largest 103 mm × 68 mm); Hb 50 g/L with fatigue and headache; no further treatment
2025-05-25	Admission due to severe fatigue. Hb 46 g/L. Ultrasound and MRI showed a large uterine mass; tumor markers normal
2025-05-25	Preoperative transfusion of 4 U packed red blood cells; Hb increased to 72 g/L
2025-05-27	Patient refused hysterectomy due to desire for fertility preservation; transabdominal myomectomy; a 13 cm × 11 cm uterine mass was resected
2025-06-06	Histopathology revealed atypical smooth muscle tumor with bizarre nuclei (STUMP); mitotic count <2/10 HPF; no necrosis. Slides reviewed by a higher-level hospital, confirming the diagnosis
2025-06-29	First postoperative follow-up ultrasound (1 month): no abnormalities detected; Hb 97 g/L (mild anemia, still below normal range)
2025-08-30	Second postoperative follow-up ultrasound (3 months): no abnormalities detected; Hb 124 g/L (normalized)
2025-12-15	Third postoperative follow-up ultrasound (6 months): no abnormalities detected. The patient remains under regular follow-up

## 3 Discussion

STUMP is a rare borderline smooth muscle tumor. The mean tumor diameter is 8 cm, and approximately 80% of patients have a tumor diameter > 5 cm (10). The clinical manifestations of STUMP are nonspecific and are similar to those of uterine leiomyoma or LMS. Common presentations include abnormal uterine bleeding, a pelvic or abdominal mass, abdominal pain or distension, anemia, infertility, or symptoms due to compression of adjacent organs (e.g., frequent urination, constipation) (1, 2, 11).

STUMP lacks specific imaging features, pelvic ultrasonography and MRI are common modalities for the diagnosis of uterine neoplasms (12). Pelvic ultrasound is often used as the first-line imaging modality for uterine tumors due to its noninvasive and radiation-free nature. However, no specific ultrasound features can reliably differentiate STUMP from uterine leiomyoma and LMS. Cotrino et al. (13) conducted a retrospective analysis and found that STUMP should be suspected when single or multiple uterine lesions exhibit the following features: isoechoic or mixed echogenicity, regular margins, absence of acoustic shadowing or calcification, internal anechoic areas or tiny cystic spaces, and detectable (minimal to abundant) blood flow signals inside and around the lesion. MRI has excellent soft-tissue resolution and is a commonly used imaging tool for evaluating uterine lesions. Studies have shown that multiparametric MRI has become the most promising diagnostic modality for differentiating leiomyomas from leiomyosarcomas. On MRI, uterine leiomyomas exhibit classic imaging features: they appear as well-circumscribed, smooth masses with homogeneous isointense or mildly hypointense signal on T1WI and characteristically low signal intensity on T2WI. DWI also shows characteristically low signal intensity, and the ADC map demonstrates correspondingly low signal. The most distinctive MRI imaging features associated with leiomyosarcoma include intermediate-to-high signal intensity on T2WI, high signal intensity on high-b-value (1000 s/mm²) DWI, accompanied by low signal intensity on ADC maps (≤ 0.905×10^-3^ mm²/s) (14, 15).

Preoperative diagnosis of STUMP is challenging, and the diagnosis relies primarily on postoperative histopathological examination. According to the 2020 World Health Organization (WHO) Classification of Tumors of the Female Genital Organs, STUMP is a diagnosis of exclusion. It is diagnosed when a smooth muscle tumor cannot be definitively classified as either a benign leiomyoma or LMS despite extensive sampling and thorough evaluation. Histologically, three main variants of STUMP are recognized: spindle-cell (also termed fusocellular), epithelioid, and myxoid. The specific diagnostic criteria are as follows (16):

For spindle-cell smooth muscle tumors, the diagnosis of STUMP can be made if any one of the following four features is present:

- Focal, multifocal, or diffuse moderate-to-severe cytological atypia with a mitotic count of 6–9/10 HPF in the absence of coagulative tumor cell necrosis;
- Definite coagulative tumor cell necrosis without cytological atypia or increased mitotic activity;
- A high mitotic count (>15/10 HPF) without coagulative tumor cell necrosis or cytological atypia;
- Diffuse moderate-to-severe cytological atypia, with the mitotic count unable to be assessed due to marked nuclear fragmentation.

The histological criteria for epithelioid and myxoid STUMPs vary considerably from those for spindle-cell STUMP.

For epithelioid smooth muscle tumors, a mitotic count of 2–3/10 HPF in the absence of coagulative tumor cell necrosis and cytological atypia is diagnostic of epithelioid STUMP.

For myxoid smooth muscle tumors, a mitotic count of 1/10 HPF in the absence of coagulative tumor cell necrosis and cytological atypia is diagnostic of myxoid STUMP.

Currently, these diagnostic criteria have been widely accepted by many scholars.

Immunohistochemistry plays an important role in the diagnosis and prognostic evaluation of uterine smooth muscle tumors that are difficult to classify. In 2019, the Italian Association of Medical Oncology (AIOM) proposed that the combined assessment of PR, p53, Ki-67, and other markers is helpful to distinguish uterine leiomyoma, STUMP, and LMS (8). In LMS, PR expression is significantly lower than in uterine leiomyoma and STUMP. p53 is highly expressed in LMS, but shows weak positivity or is negative in STUMP and leiomyoma. The Ki-67 proliferation index increases with increasing tumor aggressiveness.

Although STUMPs are slow-growing and most have a favorable prognosis, a definite risk of recurrence and malignant transformation remains. Gupta et al. (5) suggested that pathological features including marked nuclear atypia, atypical mitoses, epithelioid differentiation, vascular invasion, infiltrative growth, and ill-defined tumor borders are associated with a poor prognosis in patients with STUMP. Myxoid STUMPs, similar to their epithelioid counterparts, are associated with a higher risk of recurrence compared to conventional spindle-cell STUMPs. A retrospective analysis by Borella et al. (17) found that epithelioid features, morcellation of the myoma during surgery, and a Ki-67 index >20% were independent risk factors for recurrence and shortened recurrence-free survival. In addition, studies have shown that high expression of p53, low expression of PR, and diffuse expression of p16 are also adverse prognostic factors. Therefore, close follow-up should be performed for patients with the above pathological features (8, 18).

Surgery is the standard treatment for STUMP. Total hysterectomy with or without bilateral salpingo-oophorectomy is recommended for patients who have no desire to preserve fertility. For patients who wish to preserve fertility, tumor resection may be considered after thorough risk assessment and informed consent (19). Two pivotal studies published in 2025 have clarified the role of fertility-sparing surgery in the management of uterine smooth muscle tumors of uncertain malignant potential (STUMP). Bogani et al. (7) conducted a systematic review of nine retrospective studies comprising 327 patients, comparing outcomes after myomectomy (n=159) with hysterectomy (n=168). No significant difference in overall recurrence rates was observed between the two groups (13.2% vs 9.5%), and pregnancy was not associated with an increased risk of recurrence. Among 103 patients desiring to conceive, 40 (38.8%) achieved at least one pregnancy. Maggiore et al. (20) reported reproductive and clinical outcomes in a multicenter series of 106 STUMP patients who underwent fertility-sparing treatment. Of the 47 patients actively trying to conceive, 27 (57.4%) became pregnant, and 12 (25.5%) had more than one pregnancy. A total of 23 patients (21.7%) developed uterine recurrence, but only 2 (1.9%) experienced malignant relapse (leiomyosarcoma). Notably, a higher recurrence rate was observed among patients who became pregnant compared to those who did not (63.0% vs 7.6%). These cumulative data suggest that myomectomy may be considered a safe and effective alternative for women who wish to preserve childbearing potential, provided that the patient is fully informed of the recurrence risk, that power morcellation is avoided, and that a stringent postoperative surveillance protocol is maintained (21). An accurate assessment to exclude recurrent disease is mandatory before attempting pregnancy, and delayed hysterectomy should be recommended upon completion of childbearing.

The recurrence rate of STUMP is 7.3%–36.4% (1, 5–7), with recurrence presenting as either STUMP or LMS and involving sites such as the uterus, pelvis, retroperitoneum, lung, liver, and bone (13). Most recurrences of STUMP occur within 5 years after surgery (18). Therefore, regular follow-up for a minimum of 5 years is recommended to ensure timely detection of recurrence or metastasis. Surgery remains the first-line treatment for patients with recurrent STUMP, and the value of adjuvant hormonal therapy, radiotherapy and chemotherapy after surgery remains to be explored (7).

In the present case, a large mass of mixed echogenicity in the uterus with regular margins and no calcification or acoustic shadowing was detected by transabdominal pelvic ultrasound. MRI showed heterogeneous moderately to markedly hyperintense signal on T2WI with cystic changes. Transvaginal ultrasound could not be performed as the patient was not sexually active; therefore, characteristic ultrasound features such as internal anechoic areas or tiny cystic cavities could not be assessed in detail. In this case, the tumor was completely resected intraoperatively without morcellation, and immunohistochemistry revealed p53(-), PR(+), and a Ki-67 proliferation index of 3%–12%. Combined with the recurrence characteristics reported in the literature (8, 18), these findings suggest a relatively favorable prognosis. However, considering the risk of malignant transformation associated with STUMP, we continue to recommend that the patient undergo hysterectomy after completion of childbearing.

## 4 Conclusion

STUMP is a rare borderline smooth muscle tumor that falls between uterine leiomyoma and leiomyosarcoma, making its diagnosis particularly challenging. Through the study of this case and the relevant literature, we aim to enhance clinicians’ understanding of STUMP and provide a useful reference for preoperative evaluation.

## Data Availability

The datasets presented in this study can be found in online repositories. The names of the repository/repositories and accession number(s) can be found in the article/supplementary material.
